# Considerations regarding Maximal Lactate Steady State determination before redefining the gold‐standard

**DOI:** 10.14814/phy2.14293

**Published:** 2019-11-23

**Authors:** Ibai Garcia‐Tabar, Esteban M. Gorostiaga

**Affiliations:** ^1^ Studies, Research and Sports Medicine Center (CEIMD) Government of Navarre Pamplona Spain; ^2^ Department of Physical Education and Sport Faculty of Education and Sport University of the Basque Country (UPV/EHU) Vitoria‐Gasteiz Spain

## Abstract

We have read with interest the review written by Jones et al. (2019) published in a recent volume‐issue (volume 7, issue 5) of the journal. Criticisms regarding maximal lactate steady state intensity determination are to some extent correct and well‐justified. There are some aspects, however, that should be further clarified and documented before redefining the gold‐standard measure for the evaluation of endurance exercise capacity.

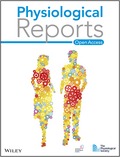

Dear Editor,

We have read with interest the review written by Jones, Burnley, Black, Poole, and Vanhatalo (2019) published in a recent volume‐issue of the journal. The review covers all the fundamental methodological factors of both maximal lactate steady state (MLSS) and critical power (CP). Criticisms regarding MLSS intensity (MLSSint) determination are to some extent correct and well‐justified. There are some aspects, however, that should be further clarified and documented before redefining the gold‐standard measure for the evaluation of endurance exercise capacity.

Historical literature reveals two classical lactate‐related thresholds considered by most exercise physiologists as the gold‐standard measures for the assessment of endurance capacity. Well before the appearance of the MLSS concept, Owles (1930) described that during constant workload tests there was a critical exercise intensity level unique to each individual above which blood lactate concentration (BLC) initiates to increase beyond resting values. In the following years this critical workload level, which always occurs at lower intensity than MLSSint and is generally termed “*Lactate Threshold* (LT)”, was widely considered as the standard criterion measure for the evaluation of endurance exercise capacity (Hollmann, 1985).

Few years later to the discovery of the “*Owles’ Point*” (Owles, 1930), i.e. the classical LT (Hollmann, 1985), and about 50 years earlier to the works of the German physiologists Mader and Heck mentioned by Jones et al. (2019), Bang (1936) demonstrated that at a given constant exercise intensity above the *“Owles’ point”* there was a second critical exercise intensity level unique to each individual indicating the highest constant exercise intensity sustainable over time without continuous BLC accumulation. The intention to verify this idea drove exercise physiologists to the creation of the MLSS concept. Therefore, the origin of the MLSS concept, although controversial, could probably be attributed to the contribution of Bang in the 30s.

Determination of classical LT and MLSS requires several constant workload tests on separate days. To overcome the shortcomings of multiple‐day testing, simpler methods have been proposed to estimate LT and MLSS from a single‐day incremental exercise test. Detailed description of LTs and ventilatory thresholds (VTs) during incremental exercise began around the 60s (1957–1964) with the works of Hollmann and Wasserman. They clearly explicated that LTs and VTs during incremental exercise were indirect methods to estimate the gold‐standard LT measured during constant exercise. Certainly, the second gold‐standard threshold (i.e. MLSS) was not clearly identifiable during incremental exercise (Hollmann, 1985). Since then, the popularity of the LTs and VTs during incremental exercise increased dramatically. Unfortunately, publishing “*new*” or “*better*” thresholds without a real scientific or on‐field practical need became, for many researchers, a popular pastime (Hollmann, 1985). This led to a considerable confusion and misinterpretation (e.g. >25 LTs), and relegated the classic gold‐standard thresholds, particularly the LT.

Despite MLSS and LT being fundamental concepts within sports medicine and exercise sciences, literature concerning their relationship is scarce. In a recent investigation (Garcia‐Tabar & Gorostiaga, 2018) we found a classical LT to be a very good MLSS predictor in male endurance‐trained runners. Classical LT corresponded to ≈77% MLSSint independent of the endurance capacity level of the runners. This suggests some degree of commonality among these two physiological parameters and advocates factors controlling LT to be partially shared with those controlling MLSS.

One of the main critique points of MLSS assessment brought by Jones et al. (2019) comes from the reliability of BLC measurement. It is well‐known that absolute BLC is influenced by the site of blood sampling, pretesting physical and hydration status, dietary or pharmacological manipulations, protocol and environmental conditions. It should be noted, however, that MLSS (and LT) assessment is based on the change of BLC (∆BLC) during exercise, rather than on absolute BLC measurements per se. Conclusions concerning the potential false positives and negatives of MLSS determination withdrawn by Jones et al. based on studies analyzing absolute BLC reliability (Bonaventura et al., 2015; Saunders, Pyne, Telford, & Hawley, 2004) seem therefore overhasty. Hauser, Bartsch, Baumgärtel, and Schulz (2013) analyzed the reliability of power and BLC at MLSS during cycling. Coefficient of variation (CV) for power at MLSS was 3%. CV of BLC at MLSS was, instead, 17%. The high day‐to‐day variability (17%) of absolute BLC at MLSS determined in this study is in line with absolute BLC reliability (11%–52%) measured in the other two studies (Bonaventura et al., 2015; Saunders et al., 2004) underlined by Jones and coauthors. MLSSint, however, was characterized by a low day‐to‐day variability (3%). In contrast to absolute BLC, ∆BLC seems appropriate and sensitive metric to enable confident assessment of MLSSint.

Precision in MLSSint determination and number of trials required is another matter that deserves further documentation. Jones et al. (2019) asserted that precision in MLSSint determination for running exercise mode is typically of 1 km h^−1^. They mentioned that precision is enhanced by the use of smaller speed differences such as 0.5 km h^−1^, “*but that this approach is likely to increase the number of trials needed for MLSS determination*.” Notwithstanding this statement was not evidence‐supported. It seems therefore worth mentioning that the review overlooked studies where MLSSint was determined with a precision of 0.2–0.35 km h^−1^ without exceeding the typical number of trials required for MLSSint determination (Garcia‐Tabar & Gorostiaga, 2018; Llodio, Garcia‐Tabar, Sánchez‐Medina, Ibáñez, & Gorostiaga, 2015; Llodio, Gorostiaga, Garcia‐Tabar, Granados, & Sánchez‐Medina, 2016). Moreover these studies demonstrated that 1–2 tests are enough to precisely estimate the MLSSint.

An advantage of MLSSint determination is the concomitant identification of the HR zone approximating the maximal metabolic steady state. This HR zone allows prescribing and monitoring training intensities relative to MLSSint. The training guidance by means of internal load seems a clinical important practical application of MLSS compared to other methods estimating the maximal metabolic steady state. A further advantage of MLSS (or LT) compared to other procedures (e.g. CP) is that it is measured using submaximal objective tests. This avoids exercising to volitional exhaustion, which depends on individual subjective motivational factors, and is many times an unfeasible procedure to test elite athletes.

A nonmentioned (Jones et al., 2019) major limitation of MLSSint assessment might be the requirement of qualified personnel for correct interpretation and appropriate handling of the results. This limitation, however, does not prevent MLSS (or LT) to continue being the classic gold‐standard metric for the evaluation of endurance exercise capacity.

## CONFLICT OF INTEREST

The authors declare no conflict of interest.
